# Group choices seemingly at odds with individual preferences

**DOI:** 10.1098/rsos.170232

**Published:** 2017-07-19

**Authors:** Michel-Olivier Laurent Salazar, Stamatios C. Nicolis, Mariano Calvo Martín, Grégory Sempo, Jean-Louis Deneubourg, Isaac Planas-Sitjà

**Affiliations:** Unit of Social Ecology, CP 231, Université libre de Bruxelles, Campus Plaine, Boulevard du Triomphe, Building NO Level 5, Brussels, Belgium

**Keywords:** social context, individual preferences, group preference, collective choice, vanillin, cockroaches

## Abstract

Numerous studies have focused on the influence of the social environment and the interactions between individuals on the collective decision-making of groups. They showed, for example, that attraction between individuals is at the origin of an amplification of individual preferences. These preferences may concern various environmental cues such as biomolecules that convey information about the environment such as vanillin, which, for some insects, is an attractant. In this study, we analysed how the social context of the cockroaches of the species *Periplaneta americana* modifies preferences when individuals are offered two shelters, of which one is vanillin scented. One of the principal results of our study is that isolated individuals stay longer and more frequently in a vanillin-scented shelter, while groups choose more frequently the unscented one. Moreover, the proportion of sheltered insects is larger when the group selects the unscented shelter. Our experimental results and theoretical model suggest that the individual preference is not inverted when insects are in a group but, rather, the response to vanillin decreases the attraction between individuals. As a result, aggregation is favoured in the unscented shelter, leading therefore to a collective inversion.

## 1. Introduction

The influence of group size and social context on different aspects of the individual has become an important issue since the pioneering works of Allee [1], Grassé [2] and Uvarov [3]. Numerous studies on various species have shown that group size can modulate the physiological and behavioural characteristics of the individuals such as the growth rate and adult weight [4–6], the individual loss of water [7,8] or the chances of escaping predators [9–11]. In recent years, the study of the influence of group size on individual behaviour during the collective decision-making has been gaining interest [12–14]. Several studies regarding model species (e.g. insects and fishes) have shown that the presence of other individuals and the attraction between them (inter-attraction) can amplify individual preferences leading to a consensus (e.g. gregariousness or trail recruitment) [15–17]. These individual preferences can even be modified by the presence of conspecifics. For example, *Perca fluviatilis* shift their levels of boldness depending on the group composition [18] and female *Xiphophorus helleri* change their male phenotype preference depending on the males' availability. Indeed *X. helleri* display a negative frequency-dependent preference for the rare-male phenotype [19]. In *Drosophila melanogaster*, both the sitter and rover strains form aggregates, but differ in their probability of entering a group and the choice is based on the number of individuals present. Philippe *et al*. [20] showed that in mixed groups, the proportion of sitter and rover individuals influenced the aggregation. Rovers (sitters) tended to behave more like sitters (rovers) when they were the minority. Finally, the increase in group size or population density gives the opportunity to exploit new resources. For example, bark beetles (*Scolytinae*) have been reported to use pheromones to mediate aggregation on trees. At low density, they attack trees that had blown over. At high density, the bark beetles still show a preference for such trees, but they are also able to aggregate on some living trees and to attack them [21,22].

Animals can display an innate preference for various food or conspecific odours [23,24]. For example, vanillin, which is present in many plants including in decaying wood, has been postulated to be a good indicator of food and is an insect attractant (including cockroaches) [25–27]. In this study, we show that groups of *Periplaneta americana* prefer a non-scented shelter over a vanillin-scented shelter, differing from isolated individuals. This result is contrary to our expectations (and differs from the above-mentioned examples), because we expected to observe a well-marked collective preference for vanillin-scented shelters as a result of the amplification of individual preferences due to the gregariousness of the cockroaches. In this paper, we test the hypothesis that the individual preference is not inverted but that the response to vanillin modulates the interactions between individuals, this modulation leading to an inversion of the preference at the collective level.

## 2. Experimental procedures

Experiments were carried out on 14 isolated males and 13 groups of 16 adult males of *P. americana* (L.) (Dictyoptera: Blattidae) in the same set-up as described in Planas-Sitjà *et al*. [28]. Only male adults were tested to exclude any behavioural variations related to the ovarian cycle [29]. This set-up included a circular arena limited by a black polyethylene ring (diameter: 100 cm, height: 20 cm) covered with a paper layer (120 g m^−2^). A lighting source was placed above the arena and provided homogeneous illumination intensity at ground level (500 ± 50 lux, with a peak at 577 nm). Two shelters made of transparent Plexiglas discs (diameter: 15 cm) were covered by a red-coloured filter film (Rosco E-Colour 19: fire), creating low luminosity zones (less than 165 lux, light spectrum above 600 nm). The centre of each disc was located 23 cm from the edge of the arena and 3 cm above the floor arena. Under one of those shelters, we placed a Petri dish with a filter paper imbued with a mix of vanillin (0.1 g l^−1^, which is according to our preliminary tests the lowest concentration inducing an individual response) and ethanol (96%) (vanillin-scented shelter; VS), and under the other shelter only ethanol (control shelter; CS). The ethanol evaporated before the trials began. Each shelter was large enough to potentially shelter all 16 cockroaches and the set-up was surrounded by white curtains to avoid spatial cues. The trials lasted 3 h and the resulting videos were recorded. The videos were analysed with the open source software USETracker (http://usetracker.org/) which allowed us to track the movement of the isolated individuals throughout the trial.

Shelter selection by cockroaches results from interactions between individuals and environmental cues [6]. In the time scale considered by our experiments, neither chemical marking nor memory effect were significant as individuals were placed in an environment free of chemical traces laid by conspecifics. The shelter use was measured by the time spent within a shelter and the number of individuals sheltering. For this reason, regarding the isolated individual trials, we focused on analysing the number of entries and their duration under each shelter. These depend on the probability of joining a shelter (i.e. number of entries/exits) and the probability of leaving it (i.e. inverse of the duration of each stay). To compare the number of entries into each shelter, we analysed the correlation between the number of entries into the vanillin-scented shelter and the number of entries into the control one. We used an extra sum-of-squares *F*-test to compare the resulting fitted slope with the hypothetical value of 1, which would indicate that both shelters are visited the same number of times [30]. We used survival curves to compare the duration of each stay in the shelters for all individuals. For the isolated males, we aggregated their dynamics (presence in the shelters) and we compared the cumulative number of sheltered cockroaches under each shelter as a function of time using two-sample Kolmogorov–Smirnov tests.

For the group trials, we used Sidak's multiple comparisons test [31] to compare the cumulative number of sheltered cockroaches under each shelter as a function of time, similar to the individual trials. The significance of statistical tests was set at *α* = 0.05. Data, statistical analyses and simulations were performed using GraphPad Prism (v. 6.04 for Windows, GraphPad Software, La Jolla, CA, USA, www.graphpad.com) and Python software (Python Software Foundation. Python Language Reference, v. 3.5.1, http://www.python.org).

## 3. Results

### 3.1. Individual preferences

**Figure 1.**
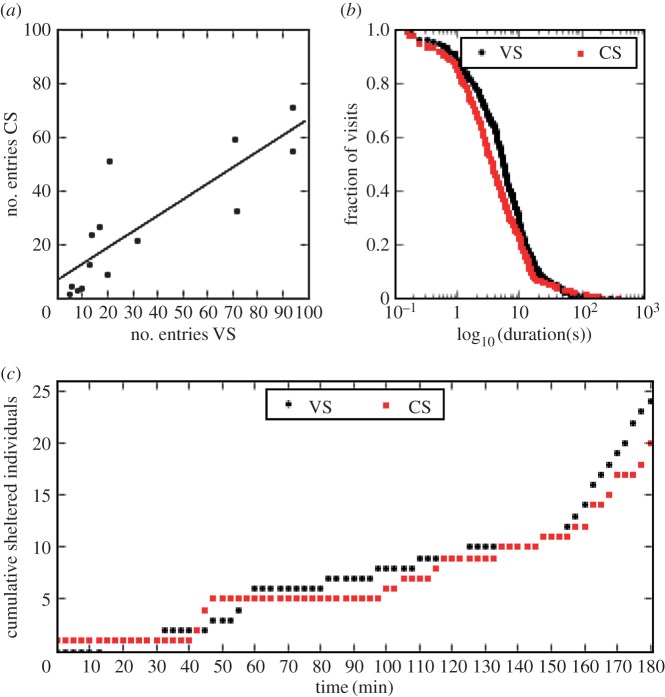
(*a*) Number of entries to the control shelter (CS) as a function of the number of entries to the VS (Number entries CS=0.6×Number entries VS+6.75; *R*^2^ = 0.71). (*b*) Survival curve of the duration (log_10_ of the time in seconds) of each stay in the VS and CS for all individuals. (*c*) Cumulative number of sheltered cockroaches under each shelter as a function of time.

We first looked at the number of entries of each isolated individual into each shelter. Figure 1*a* displays the dependence of the number of entries into the non-scented shelter (CS) as a function of the number of entries into the VS. It can be fitted with a linear relationship whose factor determines the preference for a particular shelter. For example, a slope of 1 would indicate that no preference between CS and VS is occurring. However, in our case, a slope of 0.6 (Number entries CS=0.6×Number entries VS) was observed, which indicated a preference for the VS, and that the entry probabilities were different (comparison of fits between a model with a slope of 0.6 and a model with a slope of 1: *F*-test: *F*_1,12_ = 13.92, *p* = 0.003). Moreover, the duration of the stay under the VS was longer than under the CS (mean ± s.d.: VS: 13 ± 50 s; CS: 11 ± 30 s). This difference was validated by the comparison of the survival curves of the duration of each stay of every individual in every trial (figure 1*b*). The survival curves were significantly different between VS and CS shelters (log-rank test: $\chi_{1}^{2}=7.7,$ *p* = 0.006).

Furthermore, for the isolated males (14 trials), the comparison between the cumulative number of sheltered cockroaches under each shelter as a function of time indicated that there was a tendency in favour of the VS, as seen in figure 1*c* (Kolmogorov–Smirnov test: KS statistic = 0.22, *p* = 0.05).

### 3.2. Group preferences

**Figure 2.**
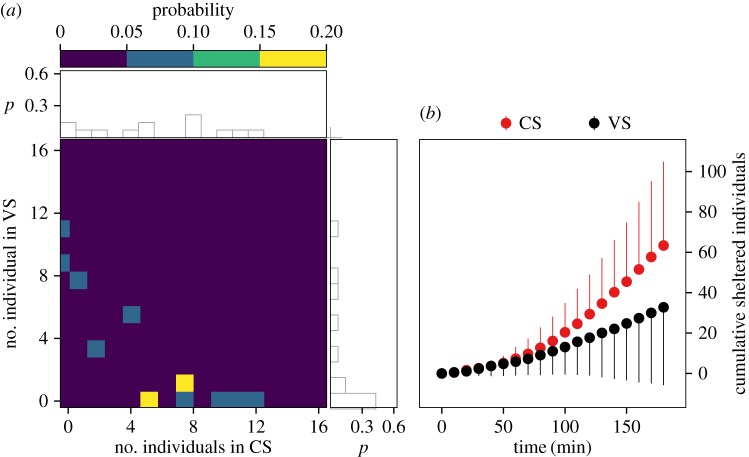
(*a*) Two-dimensional probability distributions of the number of individuals in the shelters CS and VS (coloured figure) and their projection in one dimension (CS in horizontal and VS in vertical). (*b*) Average cumulative number of sheltered cockroaches (and its standard deviation) under each shelter as a function of time.

We tested 13 groups of 16 cockroaches in the same set-up. Contrary to the isolated individuals case, the mean number of individuals in the CS was larger than in the VS (5.7 versus 2.8), while the number of experiments where the CS was selected (the number of sheltered individuals in CS was greater than in VS) was larger than for the VS (figure 2*a*). This was validated by a Sidak's multiple comparison test (from 120 min onwards, *p* < 0.05) of the cumulative number of sheltered cockroaches under each shelter as a function of time (figure 2*b*).

To test our hypothesis regarding the role of vanillin and conspecific attraction during an aggregation event, we adopted a mean field and simulation approach based on our experimental results and the model in Halloy *et al.* [32]. This experimentally validated mathematical model for collective decision-making [32–34] follows the time evolution of the fraction of individuals under each shelter as a function of key parameters such as the rates at which individuals join the shelters (*μ*_c_, *μ*_v_) and the rates at which individuals leave the shelters (*Q*_c_, *Q*_v_). The latter depends on individual leaving rates (*θ*_c_, *θ*_v_), on inter-attraction between individuals (*ρ*_c*,*_ *ρ*_v_) and on social cooperative interactions (*n*). The model was analysed to shed some light on the proximal mechanisms at the basis of the above experimental results. In particular, the model accounted for the fact that individuals explored their environment randomly [28,32], each one having a probability to encounter and join CS or VS (*μ*_c_, *μ*_v_) and a probability to leave CS or VS and start to explore (*Q*_c_, *Q*_v_).

Assuming that the sheltering time was negligible when compared with the time spent outside, *μ*_c_ (*μ*_v_) was evaluated as the mean number of entries in CS (VS) divided by the total time of the experiments (10 800 s) in the isolated individual trials (figure 1*a*; $\mu_{c}=2.5\times 10^{-3},\mu_{v}=3\times 10^{-3}$). The probabilities of joining CS or VS were assumed to be independent of the sheltered population, in agreement with previous studies [32]. *μ*_v_ was greater than *μ*_c_, corresponding to the higher attractiveness of the shelter with vanillin.

As for *Q*_c_ (*Q*_v_), previous studies [34] showed that it is related to the quality of the shelters and is dependent on the presence of conspecifics through social amplification of the duration of the stay in the shelters:

3.1*a* $$Q_{c}=\frac{\theta_{c}}{1+\rho_{c}x_{c}^{n}}$$

and

3.1*b* $$Q_{v}=\frac{\theta_{v}}{1+\rho_{v}x_{v}^{n}}.$$

Here, *θ*_c_ (*θ*_v_) depended on the characteristics of the shelter and/or the individual preferences. It can be viewed as the maximum probability of leaving the shelter CS (VS) per unit of time that occurs when a single individual is inside a shelter. The values of *θ*_c_ and *θ*_v_ were directly evaluated by the experiments. They corresponded to the inverse of the mean duration of the stay in the shelter of isolated individuals (figure 1*b*): $\theta_{c}=0.09\;s^{-1},\theta_{v}=0.07\;s^{-1}$. *x*_c_ and *x*_v_ represent the mean number of individuals in the shelters and *n* accounts for the cooperative effect of social interactions: when *n* > 1, the social interactions lead to a threshold response in the residence time as a function of conspecific presence. Based on previous evidence [32], this parameter was fixed to *n* = 2. Finally, *ρ*_c_ (*ρ*_v_) is the strength of the inter-individual attraction, given the particular shelter chosen. In our settings, we fixed *ρ*_c_ = 1, corresponding to the situation where inter-attraction between individuals is allowed in CS, and we assumed that *ρ*_v_ was lower than *ρ*_c_. Note that the parameter estimations of this study with isolated individuals are remarkably in agreement with a previous experimental analysis with two shelters identical to our control shelter (same size, same darkness) [32].

The time evolution of the mean number of individuals inside (*x*_c_, *x*_v_) and outside (*x*_e_) the shelters can therefore be written as

3.2 $$\begin{array}{rl} \frac{\text{d}x_{c}}{\text{d}t} & =\mu_{c}x_{e}-\frac{\theta_{c}x_{c}}{1+\rho_{c}x_{c}^{n}},\\ \frac{\text{d}x_{v}}{\text{d}t} & =\mu_{v}x_{e}-\frac{\theta_{v}x_{v}}{1+\rho_{v}x_{v}^{n}}\\ \;\mathrm{and}\;x_{e} & =N-x_{c}-x_{v}, \end{array}$$

where *N* is the total population.

### 3.3. Theoretical predictions and comparison with experiments

Based on the results of Dambach & Goehlen [35] on the decrease in gregariousness with increasing humidity, our hypothesis was that the interaction between sheltered cockroaches is modulated by the chemical environment, here the presence of vanillin: the inter-attraction in VS was smaller than in CS. The influence of *ρ*_v_ on the collective decision-making was therefore analysed.

**Figure 3.**
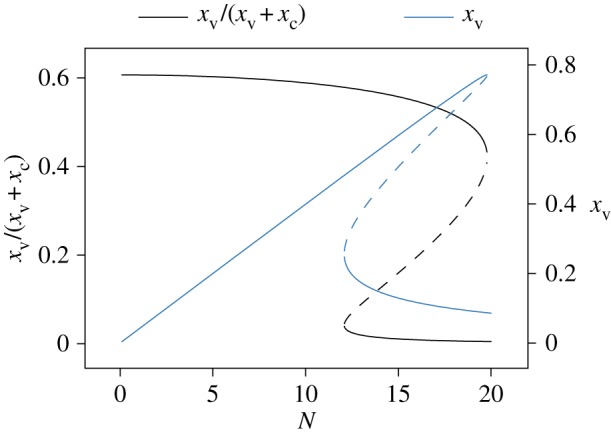
Bifurcation diagram of the steady-state solutions of the model defined by equation (3.2). *x*_v_/(*x*_v_ *+* *x*_c_) and *x*_v_ as a function of the total population *N*. The dashed line represents the unstable state. Parameter values are $\theta_{v}=23:\;33\;(\theta_{v}\text{/}\mu_{v}),\theta_{c}=36(\theta_{c}\text{/}\mu_{c}),\rho_{v}=0\text{ and }\rho_{c}=1$.

At the steady states, equation (3.2) admits up to three solutions. In the extreme case of *ρ*_c_ = 0, two of them are stable (see the electronic supplementary material). Figure 3 shows the bifurcation diagram of $x_{v}\text{/}(x_{c}+x_{v})$ (the fraction of sheltered individual in the VS as a function of the total sheltered population) and of the mean fraction numbers of *x*_v_ as a function of *N*. For small values of *N* (before the first bifurcation, at *N *≈ 12), the majority of sheltered individuals were under VS, while for large values of *N* (after the second bifurcation at *N *≈ 20), they were under CS. Surprisingly, for intermediate values of *N*, the majority of sheltered individuals could be either under VS or CS. These majorities correspond to two stable stationary solutions and between them an unstable state (dashed line, figure 3) which acts as a threshold. Note also that while the proportion of individuals under VS was larger when compared with CS for small values of *N*, the actual values of *x*_v_ were very small, in agreement with the isolated individual experiments.

**Figure 4.**
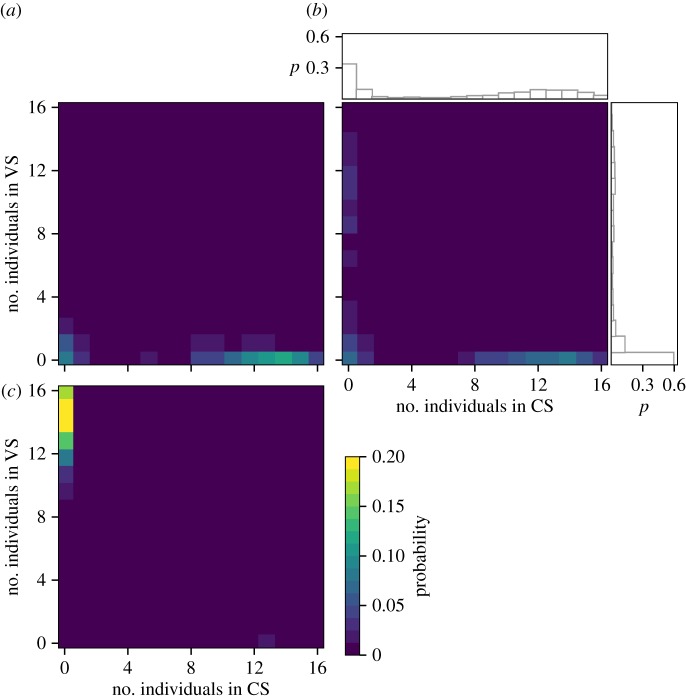
Two-dimensional probability distributions of the number of individuals in the shelters CS and VS (coloured figure) and their projection in one dimension (CS in horizontal and VS in vertical) as a result of 1000 Monte Carlo simulations. (*a*) *ρ*_v_ *=* 0, (*b*) *ρ*_v_ = 0.6 and (*c*) *ρ*_v_ = *ρ*_c_ = 1. $\mu_{c}=2.5\times 10^{-3},\mu_{v}=3\times 10^{-3}$.

To account for experimental fluctuations (which can be large because of the small number of individuals), we also performed stochastic simulations of the model (see the electronic supplementary material). Figure 4 shows the probability distributions of the number of individuals in VS and CS for three different values of *ρ*_v_ and for 1000 realizations. When *ρ*_v_ = 0 (figure 4*a*), most of the realizations ended up with the majority of individuals in CS and none in VS. For intermediate values of *ρ*_v_ (*ρ*_v_ = 0.06, figure 4*b*), more than half of the realizations ended up with the majority of individuals in CS, in agreement with the experimental results (around 60%). Finally, when vanillin does not inhibit the inter-attraction between individuals, *ρ*_v_ *=* *ρ*_c_ = 1 (figure 4*c*), almost all individuals selected systematically VS. These results show therefore that the inter-individual attraction in the VS being smaller than in the control one favours the selection of the CS, even though the VS is intrinsically (i.e. for an individual) more attractive.

**Figure 5.**
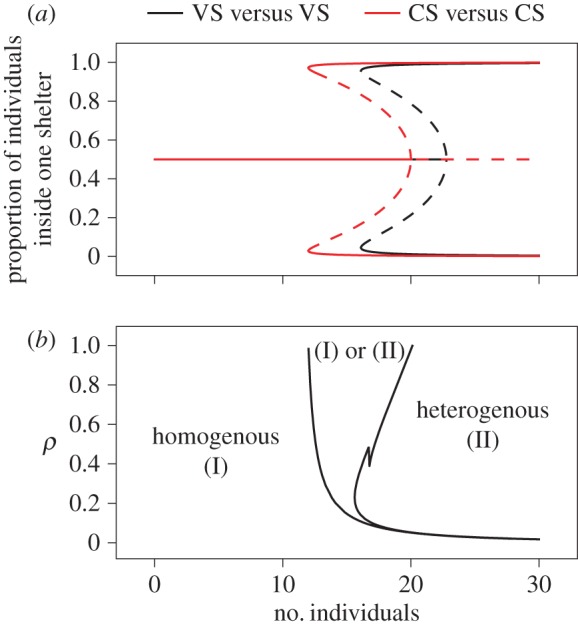
(*a*) Bifurcation diagram of the steady-state solutions of the model defined by equation (S3). *x*_1_/(*x*_1_ *+* *x*_2_) as a function of the total population *N*. The dashed line represents the unstable state for parameter values corresponding to the cases VS versus VS ($\theta_{v}\text{/}\mu_{v}=23.333$ and *ρ* = 0.6) and CS versus CS ($\theta_{c}\text{/}\mu_{c}=36$ and *ρ* = 1). (*b*) State diagram of the stable steady states of the model defined by equation (3.3) as a function of the parameters *N* and *ρ*. Other parameter values are *α* = 32.5 and *β* = 3.5.

Given the fact that our hypothesis is indeed in agreement with the results of the experiments, we go back to the ‘mean field’ model of equation (3.2) and study the symmetrical case (two control shelters or two vanillin shelters). In this case, equation (3.2) can be reduced at the steady states to an algebraic equation of degree 7 (see the electronic supplementary material). Figure 5*a* shows the bifurcation diagram of the steady states of the proportion of individuals in one shelter as a function of the total number of individuals implied in the process, for parameter values *θ* and *ρ* corresponding to two control shelters and two scented ones. The model predicts that for a small population size, individuals divide equally into the two shelters (homogeneous state). From a critical value of population, there is a region where the population either equally splits in both shelters or aggregates in a unique shelter. Finally, after a second critical value, the homogeneous state loses its stability, and only the preferential selection of a shelter is predicted. As for the differences between aggregation dynamics in the presence of two CS or two VS, we see no qualitative differences between these two situations, only quantitative ones occurring, i.e. the first bifurcation on CS appearing before the one on VS.

The hypothesis according to which a concentration of vanillin decreases the inter-attraction between individuals is now formulated in a more formal way. In the simplest scenario of a linear dependence between *θ* and *ρ*, we have:

θ=αρ+β.

Based on our experimental results, this equation assumes that for high concentration of vanillin, the inter-attraction *ρ* decreases and the intrinsic rate of leaving the shelter *θ* also decreases.

On the other side, having the experimental values of *θ*_v_ and *θ*_c_, and having inferred the values of *ρ*_v_ and *ρ*_c_, we are able to find the values of *α* and *β*, i.e.

$$\theta_{c}=\alpha \rho_{c}+\beta$$

and

$$\theta_{v}=\alpha \rho_{v}+\beta ,$$

which give *α* = 32.5 and *β* = 3.5. In a symmetrical situation, equation (3.2) then becomes

3.3 $$\frac{\text{d}x_{i}}{\text{d}t}=\left(N-\underset{i}{\sum }x_{i}\right)-\frac{(\alpha \rho +\beta )x_{i}}{1+\rho x_{i}^{2}}\;i=1,2.$$

We are therefore left with the parameter *ρ* which measures the rate at which an individual leaves a shelter, given the inter-attractivity between individuals or given the concentration of vanillin inside the shelter. Figure 5 shows the state diagram of the stable steady-state solutions of equation (3.3) (see the electronic supplementary material) as a function of *N* and *ρ*. As seen for small populations (less than 11), only the homogeneous solution is available, i.e. the population splits between the two shelters, whatever the value of *ρ* is. On the other side, for large populations, aggregation in one shelter occurs for a very wide range of *ρ* (only very small values of *ρ* lead to a homogeneous solution). In between, there is a region of uncertainty where the population either splits between two shelters or aggregates under one. This region decreases when *ρ* is decreasing, i.e. when the concentration of vanillin is increasing. We confirm once again that there are no qualitative differences between two CS (*ρ* = 1) or two VS (*ρ* = 0.6).

## 4. Discussion

In this study, we showed that preferences to settle in a vanillin-scented or an unscented shelter are modified by the social context. Isolated cockroaches tend to prefer the vanillin-scented shelter, while this preference seems inverted as far as groups of individuals are concerned. This result led us to explore the mechanisms at the origin of these differences, to develop a mathematical model in order to test a hypothesis at its origin and to provide further predictions. The hypothesis is that the concentration of vanillin within a shelter affects positively its attractivity but at the same time affects negatively the level of inter-attraction between individuals, thereby favouring their aggregation in CS. The results given by the mathematical model and its stochastic simulation version are compatible with the experiments and with the inversion of the shelter choice. Moreover, the model provides a series of predictions for different values of the total number of individuals (figure 3) and for different levels of inter-attraction (figures 4 and 5).

An alternative hypothesis would be that in the presence of conspecifics, the individual's response to vanillin decreases. In this scenario, when the attractivity of the scented and non-scented shelters are close, the mean proportion of sheltered individuals in each shelter would, however, be equal and the unscented shelter would never be preferred collectively. Another hypothesis which would give qualitatively the same results that we describe in our paper is that the presence of vanillin increases agonistic behaviours. The increase in agonistic behaviours induced by food competition is observed in several species (e.g. [36,37]). In our experiments, vanillin conveys information about food presence, but as it is lacking the odour could induce agonistic behaviours. In our set-up, we did not observe such agonist behaviours or very rarely. We explored nevertheless a model based on the agonistic behaviour (see the electronic supplementary material) and the results are in qualitative agreement with our experimental results. In the case of an experiment with two scented shelters, however, the predictions of both models concerning the influence of the size of the population on the collective patterns are ‘qualitatively different’: the model with agonistic behaviours predicts that for large populations the shelters are equally occupied, and the one with inhibition of the inter-attraction predicts that the asymmetrical distribution between shelters is maintained. Additional experiments should be carried out to validate these hypotheses.

Our experimental and theoretical results contrast with the ones found in other studies on collective choice where the increase in the number of individuals leads to an amplification of an initial preference [18–22]. Vanillin is a common natural compound produced by many plants and is attractive for some insects [25–27]. In cockroaches, vanillin is associated with food and food search, which is essentially a solitary activity [5,38]. Therefore, we hypothesize that the inhibition of social inter-attractions by vanillin and the subsequent absence of aggregate formation in the vanillin-scented shelter is a by-product of this solitary food search, the exploration being stimulated by the odour [39,40].

The inversion of preferences highlighted in this paper could also be observed in situations and gregarious species other than the cockroach–vanillin association. It is well established, for example, that humidity decreases the gregarious behaviour of cockroaches (grouping is denser under lower than under higher humidity), but individuals respond positively to the humidity [35].

Contrary to classical collective choice experiments and models showing that groups amplify the individual preferences [15–17], our model predicts that, depending on the parameter values (e.g. group size), one stimulus can lead to a collective behaviour seemingly at odds with the individual preference. It is important in this respect to carry out experiments with different odour concentrations and different population sizes to confirm our predictions as well as to further elucidate the relationship between food, odour, aggregation and its subsequent adaptive values. Further research on various environmental stimuli are needed to identify the interaction networks at the origin of different group responses.

## Supplementary Material

Detailed model and simulation description

## Data accessibility

Our data are available from the Dryad Digital Repository: http://dx.doi.org/10.5061/dryad.n6853 [41].
