# Supplementary material for: Group choices seemingly at odds with individual preferences
Source: R Soc Open Sci. 2017 Jul 19;4(7):170232. doi: 10.1098/rsos.170232 (PMC5541546; doi:10.1098/rsos.170232)
Supplement: Detailed model and simulation description [file rsos170232supp1.pdf]

Supplementary material

1 Steady states and stability

The full model of eqs. (1) and (2) can be written

$$\begin{aligned}\frac{dx_v}{dt} &= \mu_v(N - x_v - x_c) - \frac{\theta_v x_v}{1 + \rho_v x_v^2} \\ \frac{dx_c}{dt} &= \mu_c(N - x_v - x_c) - \frac{\theta_c x_c}{1 + \rho_c x_c^2}\end{aligned} \tag{S1}$$

In order to test our hypothesis, i.e.that inter-attraction between individuals is less pronounced in VS than in CS,we take the extreme case where $\rho_v = 0$. Moreover, as μ_v (μ_c) is playing basically the same role than the inverse of θ_v (θ_c) we can write at the steady state

$$\begin{aligned}0 &= (N - x_v - x_c) - \Theta_v x_v \\ 0 &= (N - x_v - x_c) - \frac{\Theta_c x_c}{1 + \rho_c x_c^2}\end{aligned} \tag{S2}$$

where $\Theta_v = \theta_v/\mu_v$, $\Theta_c = \theta_c/\mu_c$

Solving for x_c from the first equation (S2)

$$x_c = N - x_v(1 + \Theta_v) \tag{S3}$$

replacing (S3) in the second equation (S2) and rearanging, we obtain

$$\begin{aligned}&\left(\Theta_v^3\rho_c^2 + \Theta_v\rho_c^2 + 2\Theta_v^2\rho_c^2\right)x_v^3 + \left(-2\Theta_v\rho_c^2 N - 2\Theta_v^2\rho_c^2 N\right)x_v^2 + \\ &\left(\Theta_v + \Theta_v\rho_c^2 N^2 + \Theta_c + \Theta_c\Theta_v\right)x_v - \Theta_c N = 0\end{aligned} \tag{S4}$$

Eq.(S4) admits up to three solutions. We determine them as well as their stability numerically. Figure 1 displays the bifurcation diagram of $x_v/(x_v + x_c)$ and x_v as a function of N. All values of the parameters (except ρ_c) are given by the data analyses (see main text). defined

2 Monte Carlo simulations

To integrate the effects arising from the fluctuations of the dynamics, we used Monte Carlo simulations (Figure S1). The simulations were based on the differential system of equations (eqs. (2) and (S1)). The different steps can be summarised as follows: (1) initial conditions: the number of individuals in each shelter was fixed at 0 ($x_c = x_v = 0$), all individuals are outside the shelters (x_e=N) ; (2) decision process: three states are possible for each individual: outside the shelters, in shelter c or in shelter v. At each time step t, the position of each individual was checked. Then its probability of leaving the shelter C (or V)

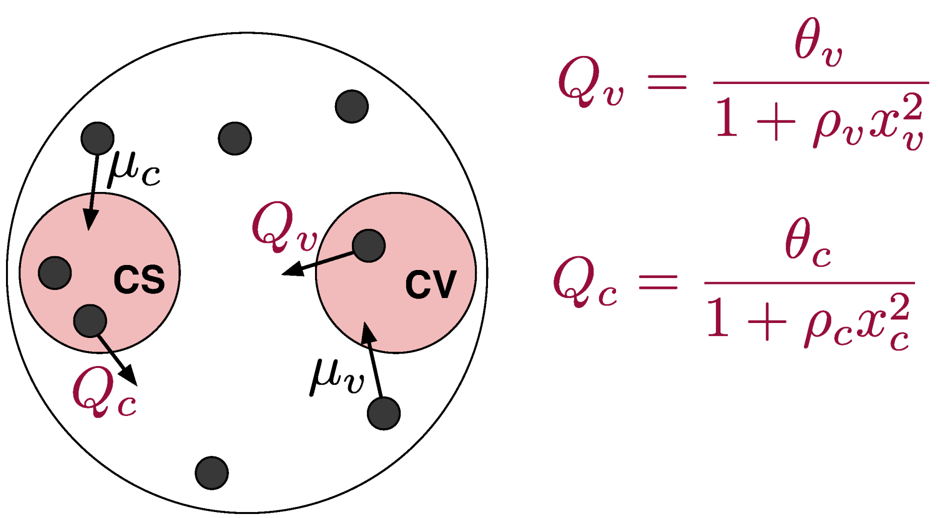

Figure S1: Schematic representation of the simulation.

and to start to explore depends on the comparison between the calculated value Q_c (or Q_v) and a random number sampled from a uniform distribution between 0 and 1. If its value is less than or equal to Q_c (or Q_v) the individual leaves (CS or VS) and goes outside. If not, it stays in shelter C (or V).

The probabilities Q_c and Q_v are updated at each simulation step in relation to the number of individuals already present in shelters.

Each exploring cockroach x_e has a probability μ_c (μ_v) to encounter and to join the site C (V). Monte Carlo simulations were run 1000 times (1000 realisations). Simulation results allowed us to follow the progress towards the stationary state on sites C and V with respect to time. The distributions of the numbers of individuals present on sites C and V at the stationary state were calculated.

Note than when both shelters are identical (e.g. two control shelters or two scented shelters), their parameters are equal.

In case of the model "agonistic behaviours" (see section 4 of the supplementary material), the probability of leaving the scented shelter Q_vs (or leaving one of the two scented shelters in case of two scented shelters) corresponds to the equation (S10)-(S11).

3 Steady states and stability of the symmetrical case

In the case of a symmetrical situation (two VS or two CS), the model is written as

$$\begin{aligned}\frac{dx_1}{dt} &= \mu\left(N - x_1 - x_2\right) - \frac{\theta x_1}{1+\rho x_1^2} \\ \frac{dx_2}{dt} &= \mu\left(N - x_1 - x_1\right) - \frac{\theta x_2}{1+\rho x_2^2}\end{aligned} \tag{S5}$$

Dividing the two equations of eqs. (S5) at the steady states and rearranging one finds

$$x_2 = x_1 = x \tag{S6a}$$

and

$$x_2 = \frac{1}{\rho x_1} \tag{S6b}$$

corresponding to the homogenous solution where individuals split in the two shelters and to the inhomogeneous solution where individuals aggregate in one of the two shelters.

Replacing eq. (S6a) into the first equation of eqs. (S5) at the steady state, we end up with the following algebraic equation for the homogenous solutions.

$$2\rho x^3 - N\rho x^2 + (2+\theta)\,x - N = 0 \tag{S7a}$$

which has up to three solutions and is resolved numerically.

Similarly, replacing eq. (S6b) into the first equation of eqs. (S5) at the steady state, we have an explicit form for the heterogenous solutions

$$\rho^2 x_1^4 - N\rho^2 x_1^3 + (\theta\rho + 2\rho) * x_1^2 - N\rho x_1 + 1 = 0 \tag{S7b}$$

which accepts upt to four solutions and can be solved numerically.

Assuming that there is a linear dependence between θ and ρ, i.e.

$$\theta = \alpha\rho + \beta$$

Eqs. (S7a)-(S7b) can be rewritten as

$$2\rho x^3 - N\rho x^2 + (2 + \alpha\rho + \beta)\,x - N = 0 \tag{S8a}$$

$$\rho^2 x_1^4 - N\rho^2 x_1^3 + (\alpha\rho^2 + \beta\rho + 2\rho) * x_1^2 - N\rho x_1 + 1 = 0 \tag{S8b}$$

Eqs. (S7a), (S7b), (S8a) and (S8b) are solved numerically and the stability of the obtained solutions are also tested numerically.

4 Alternative hypothesis : agonistic behaviours

The model that explores the alternative hypothesis of agonistic behaviours induced by vanillin is rather similar to the "social inhibition model". The rates of joining the shelters are assumed to be the same and only the rates at which individuals leave the shelters (Q_c, Q_v) are modified. In addition, we assumed that the inter-attraction between individuals is not affected by vanillin and we fixed $\rho_c = \rho_v = 1$ and in the case of the VS we added a term corresponding to the agonistic behaviour which increases with the presence of vanillin :

$$Q_c = \frac{\theta_c}{1 + \rho_c x_c^2} \tag{S9a}$$

$$Q_v = \frac{\theta_v}{1 + \rho_v x_v^2} + A_v \tag{S9b}$$

where $A_v = \epsilon_v x_v$. Here, as only vanillin induces the agonistic behaviours, the leaving rate is adjusted by a term where agonistic behaviours are proportional to the sheltered population. If the concentration of vanillin is high, ϵ_v will be larger.

The time evolution of the mean number of individuals inside ((x_c, x_v) and outside (x_e) the shelters can therefore be written as

$$\begin{aligned}\frac{dx_c}{dt} &= \mu_c x_e - \frac{\theta_c x_c}{1+\rho x_c^2} \\ \frac{dx_v}{dt} &= \mu_v x_e - \left(\frac{\theta_v}{1+\rho x_v^2} + \epsilon_v x_v\right) x_v \end{aligned} \tag{S10}$$

where $x_e = N - x_c - x_v$ and N is total population.

The steady-state solutions of eqs.(S10) are of the same nature than those of eqs. (S1), i..e., for a small number of individuals in the population, VS is preferred while for a large number ($N = 16$), CS is selected.

In the case of two identical (vanillin-scented or control) shelters, eqs.(S10) become

$$\begin{aligned}\frac{dx_1}{dt} &= \mu\,(N - x_1 - x_2) - \left(\frac{\theta}{1+\rho x_1^2} + \epsilon x_1\right) x_1 \\ \frac{dx_2}{dt} &= \mu\,(N - x_1 - x_2) - \left(\frac{\theta}{1+\rho x_2^2} + \epsilon x_2\right) x_2 \end{aligned} \tag{S11}$$

where $\epsilon = 0$ for two control shelters and $\epsilon > 0$ for two vanillin-scented ones.

Figure S2 shows the bifurcation diagram of the steady-state solutions $x_1/(x_1 + x_2)$ of eqs. (S11) as a function of N for $\epsilon > 0$ (corresponding to two vanillin-scented shelters). As seen, when N is low, the two shelters are equally occupied (homogenous solution). From a first critical value of N, the homogenous solution is still stable but coexists with the two inhomogeneous solutions (one of the two shelters is preferred). Then, after a second critical value where the homogenous solution becomes unstable (i.e., is inaccessible), only the inhomogeneous ones are available. A third critical value next occurs stabilising again the homogenous solution while the inhomogeneous solutions still exist. Finally, the inhomogeneous solutions disappear at a fourth critical value of N, where the homogenous solution exists on its own and where the population splits between the two identical shelters. The latter case is different from the social inhibition model of the previous subsections where a asymmetrical distribution of individuals between the shelters was predicted.

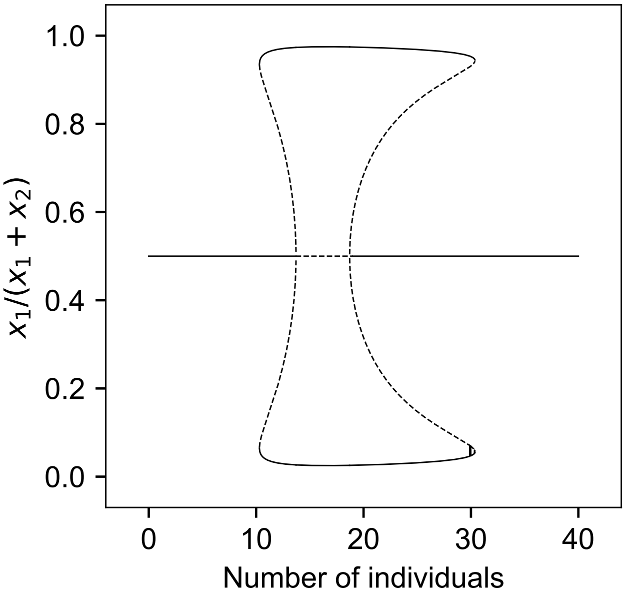

Figure S2: Bifurcation diagram of the steady-state solutions $x_{v,1}/(x_{v,1} + x_{v,2})$ of eqs. (S11) as a function of N. Parameter values are $\mu_v = 0.003$, $\theta_v = 0.07$, $\rho = 1$ and $\epsilon = 1 \times 10^{-4}$.
